# Multi-Mode Lanthanide-Doped Ratiometric Luminescent Nanothermometer for Near-Infrared Imaging within Biological Windows

**DOI:** 10.3390/nano13010219

**Published:** 2023-01-03

**Authors:** Hao Li, Esmaeil Heydari, Yinyan Li, Hui Xu, Shiqing Xu, Liang Chen, Gongxun Bai

**Affiliations:** 1 Key Laboratory of Rare Earth Optoelectronic Materials and Devices of Zhejiang Province, China Jiliang University, Hangzhou 310018, China; 2 Nanophotonic Sensors & Optofluidics Lab., Faculty of Physics, Kharazmi University, Tehran 15719-14911, Iran

**Keywords:** fluoride nanocrystals, ratiometric thermometry, lanthanide dopant, upconversion, photothermal therapy

## Abstract

Owing to its high reliability and accuracy, the ratiometric luminescent thermometer can provide non-contact and fast temperature measurements. In particular, the nanomaterials doped with lanthanide ions can achieve multi-mode luminescence and temperature measurement by modifying the type of doped ions and excitation light source. The better penetration of the near-infrared (NIR) photons can assist bio-imaging and replace thermal vision cameras for photothermal imaging. In this work, we prepared core–shell cubic phase nanomaterials doped with lanthanide ions, with Ba_2_LuF_7_ doped with Er^3+^/Yb^3+^/Nd^3+^ as the core and Ba_2_LaF_7_ as the coating shell. The nanoparticles were designed according to the passivation layer to reduce the surface energy loss and enhance the emission intensity. Green upconversion luminescence can be observed under both 980 nm and 808 nm excitation. A single and strong emission band can be obtained under 980 nm excitation, while abundant and weak emission bands appear under 808 nm excitation. Meanwhile, multi-mode ratiometric optical thermometers were achieved by selecting different emission peaks in the NIR window under 808 nm excitation for non-contact temperature measurement at different tissue depths. The results suggest that our core–shell NIR nanoparticles can be used to assist bio-imaging and record temperature for biomedicine.

## 1. Introduction

As a basic parameter, temperature has been widely observed and measured in many fields, such as bioengineering, medical treatment, and physical chemistry [1,2,3,4,5]. Ratio thermometers are based on the multiple emissions provided by phosphors, usually with significant relative sensitivity, S_r_. The dual-emission ratio luminescence thermometer shows the resolvable emissions of two different emitters and can be used as a self-reference ratio [6,7]. Temperature-sensitive signal changes can be measured using ratio intensities rather than absolute photoluminescence intensities to reduce the influence of external factors [8]. On the one hand, the high accuracy and reliability of fluorescent nanoparticles in the application of ratio optical temperature measurement can be achieved [9]. On the other hand, lanthanide-doped nanoparticles can generate upconversion luminescence under the excitation of an external excitation light source [10]. Therefore, noninvasive, non-contact biological tissue temperature measurements based on lanthanide-doped nanoparticles are possible [11].

In fact, in the field of non-contact thermometry, lanthanide-doped nanoscale temperature probes can achieve NIR emission at the same time through NIR excitation [12]. Compared with luminescence thermometers based on other materials, lanthanide-doped nanoscale temperature probes can have richer NIR emission bands by changing the doping elements, which provides more options for multi-mode temperature measurement [13,14,15]. Different from visible light, the absorption and scattering of NIR photons in in vivo environments are significantly reduced. This avoids possible tissue damage caused by UV light, providing a greater reading of tissue penetration depth and optimizing the signal-to-noise ratio [16,17]. The excellent penetrability of the NIR region to biological tissues determines its potential in biological applications, especially for the second biological window (NIR region 2). The second biological window is not only convenient for multi-mode optical measurement but also coincides with the NIR imaging window. This gives the second biological window full use of the NIR-II window (1000–1350 nm) for in vivo fluorescence imaging. This advantage is not available in conventional visible light fluorescence imaging and the NIR-I window (700–950 nm). The imaging quality is greatly improved due to low self-fluorescence and reduced scattering [18,19,20].

In this paper, the core–shell lanthanide-doped nanoparticles are designed as multi-mode NIR nanothermometers, and their applicability in temperature sensing applications is demonstrated. Compared with 980 nm, 808 nm excitation is more difficult to be scattered and absorbed by water in tumor tissues, and NIR emission involves the NIR-II window and NIR-I window [21,22,23]. Therefore, by using the two windows, not only multi-mode optical temperature measurement of tissues at different depths can be realized, but they also can be applied for deep biological NIR tissue imaging. Nanoparticles possess two optical properties, temperature-dependent thermal coupling energy level and phonon-assisted thermal sensitivity fluctuation [24,25,26]. These optical properties make Nd^3+^/Yb^3+^/Er^3+^-doped Ba_2_LuF_7_ nanocrystals have the potential to work in biological tissues as ratio optical thermometers. Their maximum thermal sensitivity is 0.63% K^−1^, which is superior to some lanthanide-doped fluoride nanoparticles and quantum dot materials, as shown in Table S1 [27,28,29]. The nanocrystals have certain absorptivity and reflectance for NIR excitation sources, as shown in Figure S1. At the same time, multiple thermal coupled energy levels can be obtained in a single probe by observing emissions in different regions and selecting different emission ratios, to realize multi-mode temperature measurement [30,31]. The novelty of this work lies in the realization of multi-mode optical temperature measurement using different NIR windows and taking into account the imaging of biological windows, which provides new support for the application of the lanthanide-doping nano platform in tumor detection and treatment.

## 2. Materials and Methods

### 2.1. Sample Fabrication

Nanocrystals with the nominal chemical formula of Ba_2_Lu_1-x-y_F_7_: zEr^3+^/yYb^3+^ xNd^3+^ were prepared from trifluoroacetate materials. The values of x, y, and z are 0.03, 0.24, and 0.03, respectively. The precursor of lanthanide trifluoroacetic acid was prepared from the corresponding lanthanide and BaCO_3_ and trifluoroacetic acid (99%). The raw materials were composed of Lu_2_O_3_, BaCO_3_, Er_2_O_3_, Nd_2_O_3_, Yb_2_O_3_, and La_2_O_3_. The purity of the above oxides is 99.99%. The nanomaterials were fabricated by thermal decomposition. In the case of the Ba_2_LuF_7_: 3%Nd^3+^, 24%Yb^3+^, 3%Er^3+^ triple-doped samples, 0.015 mmol of Er_2_O_3_, 0.35 mmol of Lu_2_O_3_, 0.12 mmol of Yb_2_O_3_, and 0.015 mmol of Nd_2_O_3_ were dissolved to transparency in 10 mL trifluoroacetic acid solution, where the concentration of the trifluoroacetic acid solution was 50% at 105 °C. The temperature rose to 120 °C and slowly evaporated the remaining water and acid until it left a dry solid. After that, the trifluoroacetate was mixed with 11 mL octadecene and 7 mL oleic acid in a three-necked flask. The purity of oleic acid and octadecene here is 90%. The solution was heated to 130 °C in a nitrogen-filled flask and stirred quickly for 90 min to remove the remaining oxygen and water. It was then heated to 300 °C under dry nitrogen at 10 °C/min and kept at this temperature for 2 h. All the above chemical materials were obtained from Aladdin in Shanghai.

### 2.2. Characterization of Sample

The X-ray diffraction (XRD) data were collected by X-ray diffractometer (D2 PHASER, Bruker, Germany) with Cu-Kα (1.5406 Å) radiation. The elemental composition of nanocrystals was investigated by X-ray photoelectron spectroscopic analysis (XPS, K-Alpha, Thermo Scientific, WWLP, USA). The surface morphology and particle size distribution of nanocrystals were recorded by a transmission electron microscope (TEM, Tecnai G2 F20, FEI, OR, USA). The optical temperature measurement platform built by us not only recorded the temperature detection performance of the prepared nanocrystals but also evaluated their photothermal performance. The fluorescence spectrophotometer type QM8075-11(HORIBA, Japan) was used to record the photoluminescence spectra of nanomaterials. The ultraviolet absorption spectrum and Fourier transform infrared spectrum of nanoparticles were obtained by an ultraviolet spectrophotometer (UV-3600, SHIMADZU, Japan) and a Fourier transform infrared spectrometer (iS50, Nicolet, WI, USA), respectively. The NIR imaging of nanoparticles was performed by a NIR camera in a dark chamber (3200 OV1080P, Angeleyes, ShengZhen, China). In the absence of additional instructions, the above experiments were carried out at room temperature.

## 3. Results and Discussion

### 3.1. Phase Structure and Morphology of Ba_2_LuF_7_@Ba_2_LaF_7_ Nanoparticles

**Figure 1:**
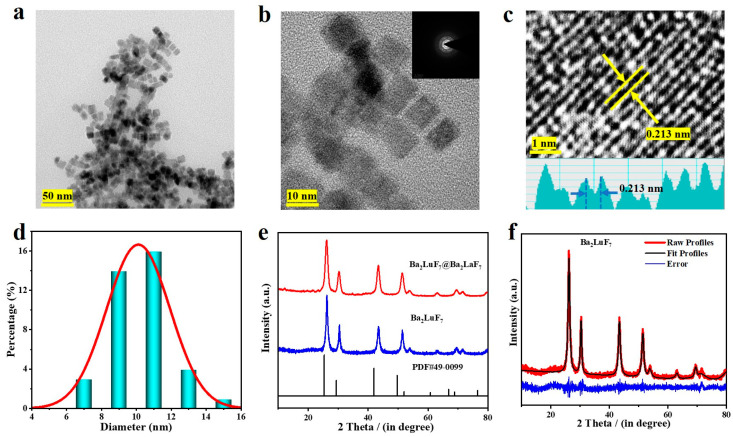
(**a**) Morphological characterization of nanocrystals at 50 nm scales was obtained by TEM. (**b**) Morphological characterization of nanocrystals at 10 nm scales was obtained by TEM. The selective area electron diffraction (SAED) pattern is presented in the illustration on the upper right. (**c**) The high-resolution TEM (HR-TEM) image of Ba_2_LuF_7_@Ba_2_LaF_7_. (**d**) The particle size distribution plot for Ba_2_LuF_7_@Ba_2_LaF_7_. (**e**) XRDs of tri-doped Ba_2_LaF_7_ and Ba_2_LuF_7_@Ba_2_LaF_7_ nanocrystals were compared with those of standard cards. (**f**) Rietveld XRD refinement for Ba_2_LuF_7_@Ba_2_LaF_7_ nanocrystals.

The morphology and size of the compound were measured by transmission electron microscope (TEM). As demonstrated in Figure 1a,b, the Ba_2_LuF_7_@Ba_2_LaF_7_ nanocrystals were composed of a large number of cubic phase nanoparticles, about 10 nanometers in length. Figure 1a–c show the morphological characterization of nanocrystals at different scales. Based on 100 nanoparticles, Figure 1d also reveals that synthesized Ba_2_LuF_7_@Ba_2_LaF_7_ nanoparticles were uniformly sized with mean particle sizes of 10 ± 2 nm. Moreover, the structure of the synthesized nanocrystals was confirmed by X-ray diffraction (XRD) in Figure 1e. According to the half-width of the three strongest diffraction peaks of the XRD pattern, the average diameter of the nanoparticles was about 12.7 nm, which was calculated by the Debye–Scherrer formula. The calculated results were found to be close to the TEM analysis. The XRD patterns for the synthesized Ba_2_LuF_7_@Ba_2_LaF_7_ nanoparticles (Figure 1a) confirmed that the samples are crystalline with the diffraction peaks indexed to Ba_2_LaF_7_ (PDF # 49-0099). The diffraction peak of the Ba_2_LuF_7_@Ba_2_LaF_7_ nanoparticles was slightly offset from the Ba_2_LaF_7_ (PDF # 49-0099), which was due to the substitution of La^3+^ ions by Lu^3+^ ions in the lattice position, forming the cubic phase Ba_2_LuF_7_. For the sake of deeply comprehending the impact of the Yb^3+^/Nd^3+^/Er^3+^ ions doping on the crystal structure of the studied samples, the Rietveld refinements of the typical Ba_2_LuF_7_: Yb^3+^/Nd^3+^/Er^3+^@Ba_2_LaF_7_ nanoparticles based on their XRD data were carried, as depicted in Figure 1f. As expected, these calculated diffraction bands were identical to those of the experimental data, which implied that the resultant nanoparticles exhibited a pure cubic phase [32]. As shown on the upper right in Figure 1c, the high-resolution TEM image consisted of clear lattice fringes with a spacing of around 0.213 nm.

### 3.2. XPS Analysis of Ba_2_LuF_7_ and Ba_2_LuF_7_@Ba_2_LaF_7_ Nanoparticles

The chemical composition and electronics state information of Ba_2_LuF_7_ and Ba_2_LuF_7_@Ba_2_LaF_7_ nanoparticles were investigated by X-ray photoelectron spectroscopic (XPS) analysis. Figure S2a,b describe the recorded XPS spectra of Ba_2_LuF_7_ and Ba_2_LuF_7_@Ba_2_LaF_7_ nanoparticles, respectively. In addition, these XPS survey spectrum peaks verified the presence of Ba, Lu, La, and F aspects. Additionally, the C1s peak located at a binding energy of 285 eV might be due to the XPS instrument’s adventitious hydrocarbon. In Figure S2c, The Ba 3d deconvoluted spectrum shows the binding energy peak, and 780 eV for Ba 3d spin-orbit components is fairly similar to the theoretical value of Ba^2+^. Figure S2d–f core level spectra show the peaks on La 3d, Lu 4d, and F 1s at 836, 198, and 684 eV, respectively [33]. This proved the existence of Ba_2_LuF_7_ and Ba_2_LaF_7_. Therefore, the XPS studies confirmed that Ba_2_LuF_7_@Ba_2_LaF_7_ nanoparticles were obtained without foreign contaminants.

### 3.3. Photoluminescence Properties of Nanoparticles

**Figure 2:**
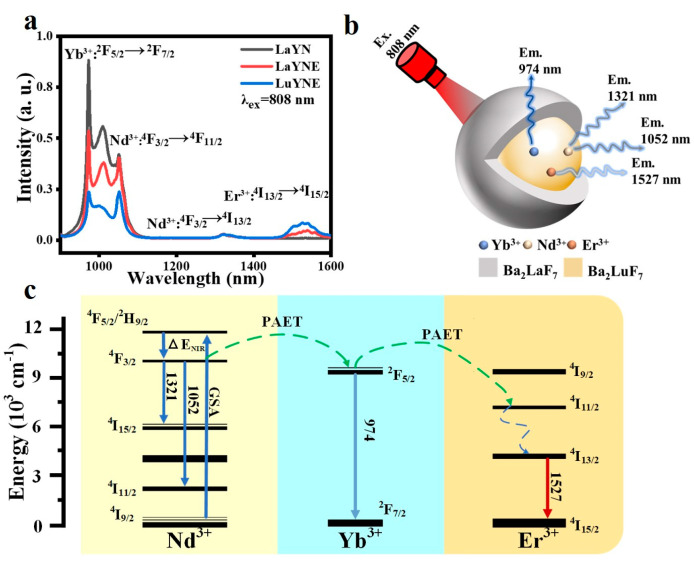
(**a**) The emission spectra of Yb^3+^/Nd^3+^ and Yb^3+^/Er^3+^/Nd^3+^ codoped Ba_2_LaF_7_ and Ba_2_LuF_7_ nanoparticles, and the excitation light source wavelength is 808 nm. (**b**) Schematic illustration of the luminescent nanoparticles excited by 808 nm laser. (**c**) General energy level diagram of Er^3+^, Yb^3+^, and Nd^3+^ ions.

We investigated the near-infrared upconversion photoluminescence properties of the Ba_2_LuF_7_: Nd^3+^ /Er^3+^/ Yb^3+^ system based on the spectral data of the nanoparticle system. The photoemission spectrum of the prepared tri-doped Ba_2_LuF_7_ nanoparticles under 808 nm photoexcitation is shown in Figure 2a. The curve shown in Figure 2a shows that the upconversion emission performance of all samples is consistent with the reported performance. It consists of four strong peaks triggered by the Yb^3+^/Er^3+^/Nd^3+^ transition, and the central wavelengths are 974 nm, 1052 nm, 1321 nm, and 1527 nm, respectively. These transitions are reported to correspond to the ^2^F_5/2_→^2^F_7/2_ transition of the Yb^3+^ ion. This proves that Yb^3+^: ^2^F_5/2_ level populations can be reached from Nd^3+^ ions to Yb^3+^ ions through phonon-assisted energy transfer (PAET) [34,35]. Furthermore, the PAET process between Er^3+^ and Yb^3+^ can be viewed as a ^4^I_13/2_→^4^I_15/2_ transition, because the emission of Er^3+^ can also be found in the fluorescence spectrum of tri-doped Ba_2_LuF_7_ nanoparticles. In addition, the emission of Ba_2_LuF_7_: Yb^3+^/Nd^3+^/Er^3+^ (1052 nm and 1321 nm) in the near-infrared region matches the ^4^F_3/2_→^4^F_11/2_ and ^4^F_3/2_→^4^I_13/2_ transitions of Nd^3+^ ions [36,37]. Finally, the transitions of Yb^3+^, Nd^3+^, and Er^3+^ marked in Figure 2c are distributed in the NIR region of NIR-I, NIR-II, and NIR-III.

In addition, it was found that both green and red upconversion emission intensities were sharply increased by enhancing the excitation optical power, and that the slopes of the emission peaks obtained by linear fitting were 1.213, 1.166, and 1.160, respectively.

Figure S3a,b show that these transitions correspond to two photons, which is beneficial in reducing the energy loss caused by scattered light, thus facilitating fluorescence imaging [38]. Similarly, the same pattern applies to launches located at NIR-I and NIR-II. Notably, the coating of a Ba_2_LaF_7_ shell onto Ba_2_LuF_7_: Yb^3+^/Nd^3+^/Er^3+^ nanoparticles enhanced the upconversion luminescence intensity 3.5-fold (Figure S3c,d). These results suggest that Ba_2_LaF_7_ shell passivation can effectively mitigate the quenching of trapped upconversion luminescence on nanocrystal surfaces.

### 3.4. Sensitivity of Ratiometric Thermometry for Nanoparticles

**Figure 3:**
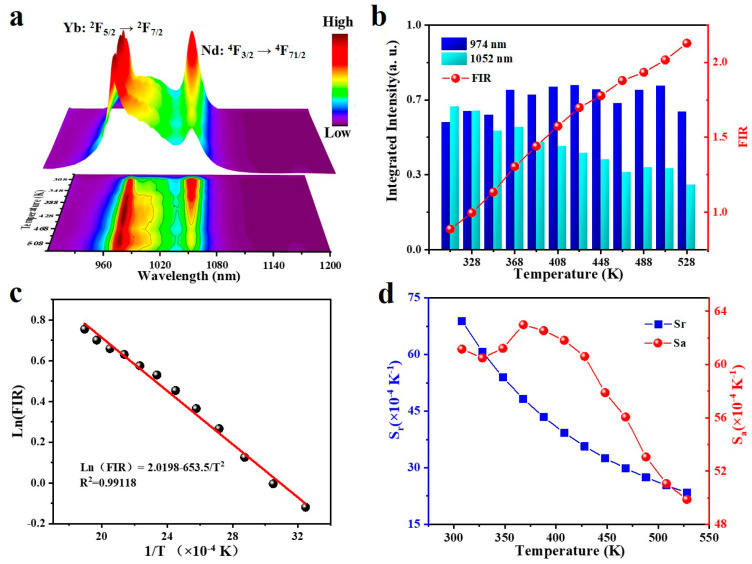
(**a**) Photoluminescence spectra curve of the Ba_2_LuF_7_: Yb^3+^/Nd^3+^/Er^3+^ nanoparticles covering 308–528 K temperature range. Integrated upconversion intensities at 974 nm and 1052 nm. (**b**) Temperature dependence of the fluorescence ratio values of thermally coupled energy levels. (**c**) Ln(FIR) is an inverse absolute function of absolute temperature. (**d**) The temperature function of the thermal coupling level of nanomaterials based on the fitting of Sa and Sr values.

The upconversion emission spectra of nanoparticles prepared in this study were fitted as temperature-dependent functions to verify their feasibility as optical thermometers under the premise of temperature control in the 308–528 K range. As disclosed in Figure 3a, the upconversion emission intensity of Ba_2_LuF_7_: Yb^3+^/Nd^3+^/Er^3+^ nanoparticles depends on temperature. Figure 3a specifically shows that the upconversion emission intensity corresponding to the Nd^3+^ ion shows an opposite trend with the increase in temperature, which is triggered by the thermal quenching effect, according to common reports. However, from the integrated upconversion intensities spectral curve at different room temperatures (Figure 3b), the emission intensity of the ^2^F_5/2_→^2^F_7/2_ transition increases slowly compared with that of the ^4^F_3/2_→^4^F_11/2_ transition due to the phonon auxiliary heat of the Nd^3+^ phonon. After it is verified that the FIR values of the two kinds of emissions depend on temperature, it can be predicted that the nanoparticles prepared in this work have the potential to be used in optical temperature sensors. Compared with the thermal coupling energy levels of 540 and 521 nm in Figure S3, the corresponding energy level emissions of 974 and 1052 nm also accord with the Boltzmann factor to control the thermal balance, which can be described as the following:

(1) $$FIR=\frac{I_{974}}{I_{1052}}=C\exp\left(\frac{-\Delta E}{kT}\right)$$

*K* and *T* correspond to the Boltzmann constant and Kelvin scale temperature, respectively; *C*, as a constant, is related to the choice of the main material; ΔE is determined by the energy gap between the ^4^F_3/2_ and ^2^F_5/2_ states. As disclosed in Figure 3b, the relative value of *I*_1052_ weakened steadily. Meanwhile, the decline of *I*_1052_ exceeds that of *I*_974_. *I*_974_/*I*_1052_ reveals an escalating trend from 0.88775 to 2.12769, because the downward trends in *I*_974_ and *I*_1052_ were different.

The absolute temperature (1/*T*) and ln(*I*_974_/*I*_1052_) were linearly fitted according to Equation (1). This is an inverse curve with a regression coefficient (R^2^) of 0.99829, and all points could pass through the fitting line, as disclosed in Figure 3c. The relevant parameters of the fitted curve are −0.06535 and 2.0198, respectively, corresponding to the slope and intercept. The fitting curve of Ln(*I*_974_/*I*_1052_) and 1/*T* has a good linear trend in the temperature range of 308 K to 528 K.

As an important index to measure optical temperature sensitivity, S_a_ represents absolute sensitivity and *S_r_* represents relative sensitivity. The value of *S_a_* can be calculated by the following formula:

(2) $$S_{a}=\left|\frac{dFIR}{dT}\right|=FIR\left(\frac{\Delta E}{kT^{2}}\right)$$

Figure 3d shows the function curve of *S_a_* and *S_r_* from 308 to 548 K concerning temperature. Since the slope and intercept of the fitted line were obtained in Figure 3c, the values of *S_r_* and *S_a_* can be obtained only by substituting them into Equations (3) and (2). As disclosed in the figure, due to the influence of the auxiliary heat of the Nd^3+^ ion phonon, the greatest *S_a_* first occurs when the curve is at 368 K, which is 0.50% K^−1^, and then it gradually decreases as the temperature goes up. When the temperature goes up, *S_r_* decreases gradually. At this point, the greatest value is 0.63% K^−1^ at 308 K.

As an important parameter to measure the performance of optical thermometers, the relative temperature can be calculated by the following formula:

(3) $$S_{r}=\left|\frac{1}{\;\mathrm{FIR}\;}\frac{dT}{\;\mathrm{dFIR}\;}\right|\times 100\%$$

**Figure 4:**
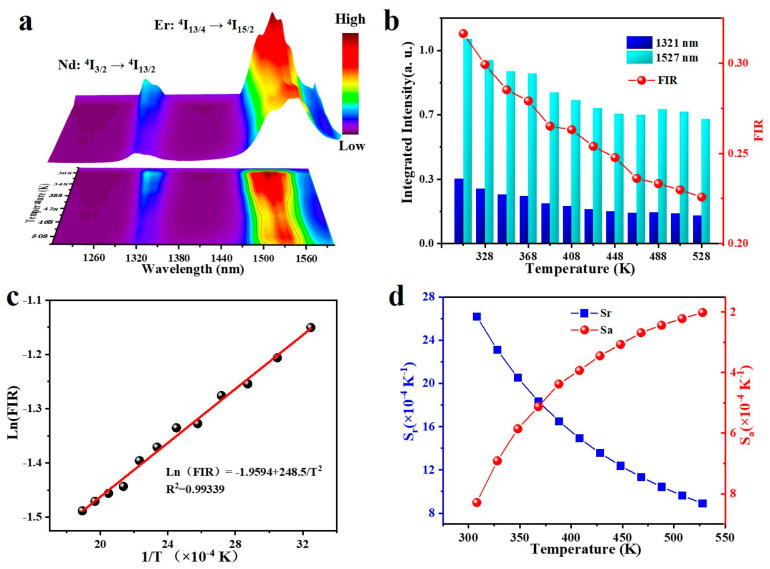
(**a**) Photoluminescence emission spectra curve of the Ba_2_LuF_7_: Nd^3+^/Yb^3+^/Er^3+^ nanoparticles covering 308–528 K temperature range. Integrated upconversion intensities at 1321 nm and 1527 nm. (**b**) Temperature dependence of the fluorescence ratio values of thermally coupled energy levels. Dependence of FIR values of the (**b**) thermally coupled levels on temperature. (**c**) Ln(FIR) as an inverse absolute function of inverse absolute temperature. (**d**) The temperature function of the thermal coupling level of nanomaterials based on the fitting of *S_a_* and *S_r_* values. *S_a_* and *S_r_* values are based on the (**d**) thermally coupled levels as a function of temperature.

Because a certain amount of Nd^3+^ ions were added into the prepared Yb^3+^/Er^3+^ nanocrystals in this work, a new emission peak in the near-infrared region of the excitation spectrum could be observed. The nanocrystals were exposed to an 808 nm light source. For instance, the peak of the spectrum at 1321 nm is the energy level transition of Nd^3+^: ^4^*I*_3/2_→^4^*I*_15/2_. It can be understood from the revelation in Figure 4a that the upconversion luminescence intensity declines with the rise in temperature, within the range of 308 ~ 548 K. Similarly, due to the phonon-assisted heat of Er^3+^ phonons, the emission intensity of the ^4^*I*_13/4_→^4^*I*_15/2_ transition fluctuates with the rise and fall of the experimental temperature. Figure 4b shows a comparison of the luminescence intensity from 308 to 548 K with temperature changes at 1321 nm and 1527 nm wavelengths. As mentioned above, even though the luminescence intensity corresponding to the 1321 nm level declines with the rise in temperature, the luminescence intensity corresponding to the 1527 nm level decays faster under the same conditions. This will cause the FIR value of *I*_1321_/*I*_1527_ to decline when the temperature rises. It has the greatest FIR of 0.31652 at 308 K and the least FIR of 0.22582 at 548 K.

As shown in Figure 4c, Ln(FIR) was linearly fitted to 1/*T* by Equation (1) to obtain a line with R^2^ = 0.99339. According to the fitting results, the calculated Ln(FIR) value coincides with the fitting line. The relevant parameters of the fitted curve are 0.2465 and −1.9594, respectively. Figure 4d reveals the curve composed of the values of *S_a_* and *S_r_* calculated by substituting them into Equations (2) and (3). In the range of 308 K to 548 K, *S_a_* rose with the rise in temperature, and the greatest value was 0.08% K^−1^. The variation trend of the *S_r_* value was opposite to that of *S_a_*, and the greatest value was 0.262% K^−1^.

### 3.5. Fluorescence Imaging of Nanoparticles in Biological Windows

According to the above data, nanoparticles have the potential to be applied in multi-mode fluorescence temperature measurement, which provides the possibility for the universality of non-contact tissue temperature measurement. As shown in Figure S4, the non-contact optical temperature measurement system simulates biological tissues at different depths by controlling lipid thickness. Nanoparticles are injected into biological tissues. The light received under the excitation of the 808 nm external excitation light source more easily penetrates the NIR region of biological tissues than the visible light spectrometer. The NIR region used by different temperature measurement modes in this work can be found in Figure S5. These thermometric models are based on the energy transfer process between Yb^3+^/Nd^3+^/Er^3+^. Nd^3+^ ions in the first region and the second region are used as the selector switch to determine the fluorescence ratio temperature measurement for narrowband separation and broad-band separation, respectively. The fluorescence ratio thermometry of mode-two width separation can detect deeper tissues thanks to the excellent penetration of NIR region 2, and the fluorescence ratio thermometry of mode-one narrow separation has higher sensitivity.

**Figure 5:**
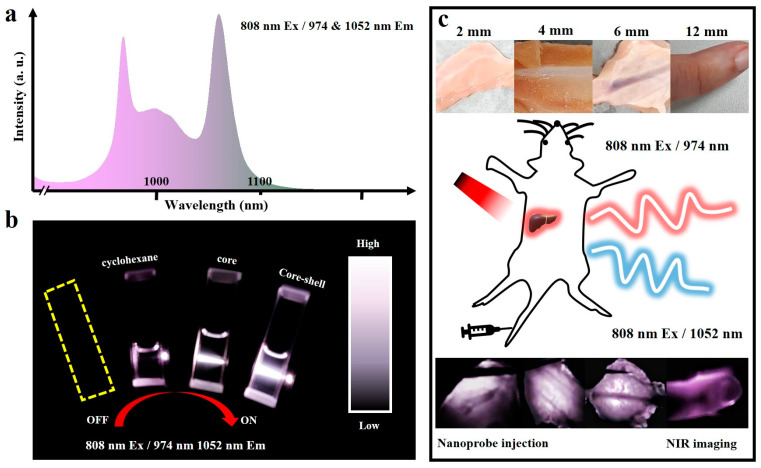
(**a**) The selected NIR imaging interval. (**b**) NIR luminescence of nanoparticles. (**c**) Schematic of biological tissue imaging of nanoparticles.

NIR window luminescence has low water absorption efficiency and good tissue penetration, which is often used in biological tissue imaging [39,40,41,42]. Taking the two emissions of region 1 and region 2 in mode 1 as an example (Figure 5a), it can be noticed that core–shell nanoparticles in the darkroom under the NIR source glow brightly and clearly in the field of view of the infrared camera (Figure 5b). As shown in Figure 5c, chicken tissues with thicknesses of 2 mm, 4 mm, and 6 mm were selected for imaging. In the image of the 2 mm tissue there are shaded areas, which are attached bone sheets on the back of the biological tissue. This indicates that the NIR emission of nanocrystals has different transmittance to different tissue components, which makes it possible to image foreign bodies in tissues. In 4 mm and 6 mm tissue imaging, more obvious cartilage tissue and ribs can be observed, which further confirms the imaging ability of nanocrystals. In addition, the addition of 12 mm human pinky finger imaging still allows relatively clear observation of bones and joints, indicating the potential of nanocrystals for deep tissue imaging. Furthermore, image quality improvement combined with algorithm image processing can further improve image clarity to achieve auxiliary tumor tissue and blood vessel imaging, which reflects the potential of nanocrystals in fine tissue imaging. Hence, the nanoparticles are injected into mice after being biologically modified to help image specific tissues and organs through the mice’s circulatory system. As for multi-mode fluorescence ratio temperature measurement, the receiving window can be selected according to the depth of the desired imaging location due to the stronger tissue penetration of the NIR second region window. Taken together, these results finally show that our nanocrystals have some possibility for imaging in biological windows.

## 4. Conclusions

All in all, we worked on the non-contact multifunctional biological applications of lanthanide-doped nanoparticles based on NIR luminescence. On this basis, the advantages of FIR optical thermometry technology were shown, and the maximum S_r_ is close to 0.63% K^−1^ in the range of 308–538 K. The FIR temperature measurement mode can be selected according to the tissue depth of the temperature measurement site. The flexible selection broadens the application scenario and gives broad application prospects in the field of optical thermometry. At the same time, the NIR imaging of the material reveals that the temperature-assisted imaging of the material works well in practical applications. In the end, the lanthanide-doped nanoparticles designed in this paper have potential applications in NIR luminescence-assisted imaging and multi-mode optical temperature measurement of biological tissues.

## Supplementary Materials

The following supporting information can be downloaded at: https://www.mdpi.com/article/10.3390/nano13010219/s1, Table S1. Comparison of optical properties of some nanomaterials and their applications in proportional nanothermometers; Figure S1. (a) UV absorption spectra of nanoparticles in cyclohexane. (b) Fourier infrared spectroscopy of nanoparticles; Figure S2. (a,b) XPS survey spectra of the Ba2LuF7: Yb3+/Nd3+/Er3+ and Ba2LuF7: Yb3+/ Nd3+/Er3+@Ba2LaF7 nanoparticles, respectively. And deconvoluted spectra of (c) Ba 3d, (d) La 3d, (e) Lu 4d, and (f) F 1s region of Ba2LuF7@Ba2LaF7; Figure S3. (a) Upconversion luminescence spectra in visible regions of nanocrystals excited at different power densities of 980 nm. (b) The relationship between emission intensity and excitation power. (c,d) Comparison of upconversion luminescence intensity between core and core-shell nanoparticles; Figure S4. Diagram of the non-contact temperature measuring system; Figure S5. Schematic diagram of multi-mode temperature measurement principle.
