# Supplementary material for: Multi-Mode Lanthanide-Doped Ratiometric Luminescent Nanothermometer for Near-Infrared Imaging within Biological Windows
Source: Nanomaterials (Basel). 2023 Jan 3;13(1):219. doi: 10.3390/nano13010219 (PMC9824890; doi:10.3390/nano13010219)
Supplement: Supplementary file 1 [file nanomaterials-13-00219-s001.zip › nanomaterials-2120515-supplementary.pdf]

Supporting Information

Multi-Mode Lanthanide-Doped Ratiometric Luminescent Nanothermometer for Near-Infrared Imaging within Biological Windows

Hao Li [1], Esmaeil Heydari [2], Yinyan Li [1,*], Hui Xu [1], Shiqing Xu [1], Liang Chen [1,*] and Gongxun Bai [1,*]

1 Key Laboratory of Rare Earth Optoelectronic Materials and Devices of Zhejiang Province, China Jiliang University, Hangzhou 310018, China
2 Nanophotonic Sensors & Optofluidics Lab, Faculty of Physics, Kharazmi University, Tehran 15719-14911, Iran
* Correspondence: liyinyan@cjlu.edu.cn (Y.L.); lchen@cjlu.edu.cn (L.C.); baigx@cjlu.edu.cn (G.B.)

.

Table S1. Comparison of optical properties of some nanomaterials and their applications in proportional nanothermometers.

Sensing materials	Temperature range	Sensor sensitivity	Reference
CdTe	80-360 K	0.007	[27]
CdSe quantum dots	300-323 K	0.0069 K^{-1}	[28]
$NaBiF_4$:Yb^{3+}/Er^{3+}/20%Gd^{3+}	148-498 K	0.0037 K^{-1}	[29]
Ba_2LuF_7:Er^{3+}/Yb^{3+}/Nd^{3+}	308-548 K	0.0063 K^{-1}	This work

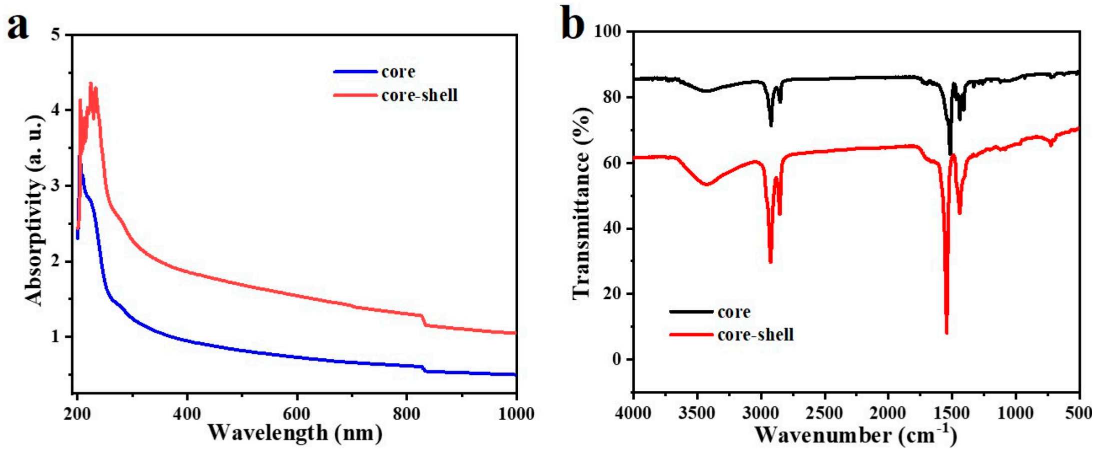

Figure S1. (a) UV absorption spectra of nanoparticles in cyclohexane. (b) Fourier infrared spectroscopy of nanoparticles.

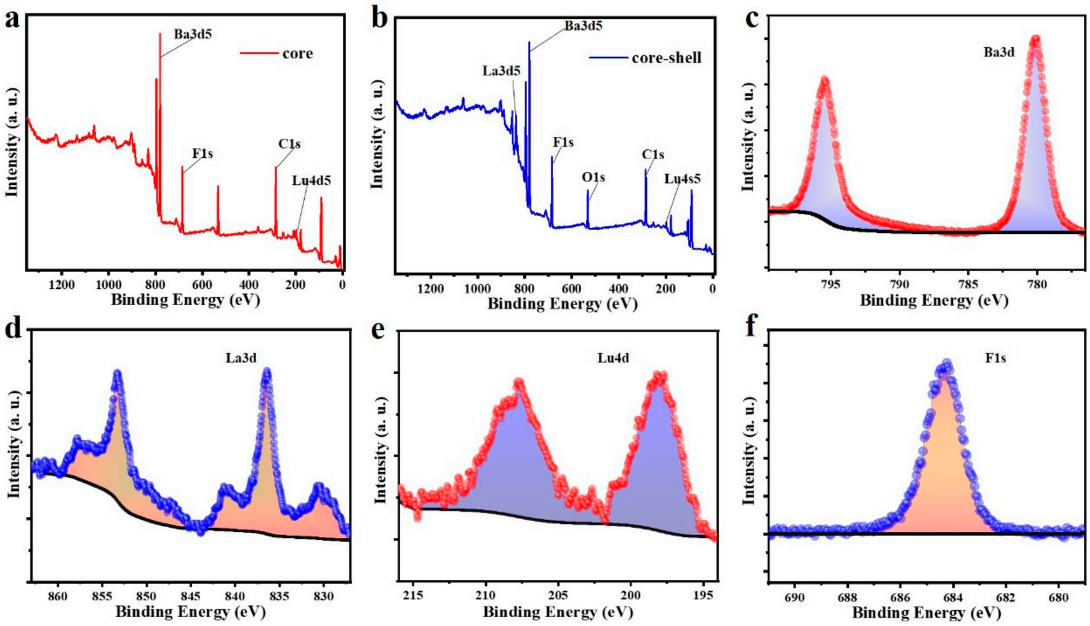

Figure S2. (a,b) XPS survey spectra of the Ba_2LuF_7: Yb^{3+}/Nd^{3+}/Er^{3+} and Ba_2LuF_7: Yb^{3+}/ Nd^{3+}/Er^{3+}@Ba_2LaF_7 nanoparticles, respectively. And deconvoluted spectra of (c) Ba 3d, (d) La 3d, (e) Lu 4d, and (f) F 1s region of Ba_2LuF_7@Ba_2LaF_7.

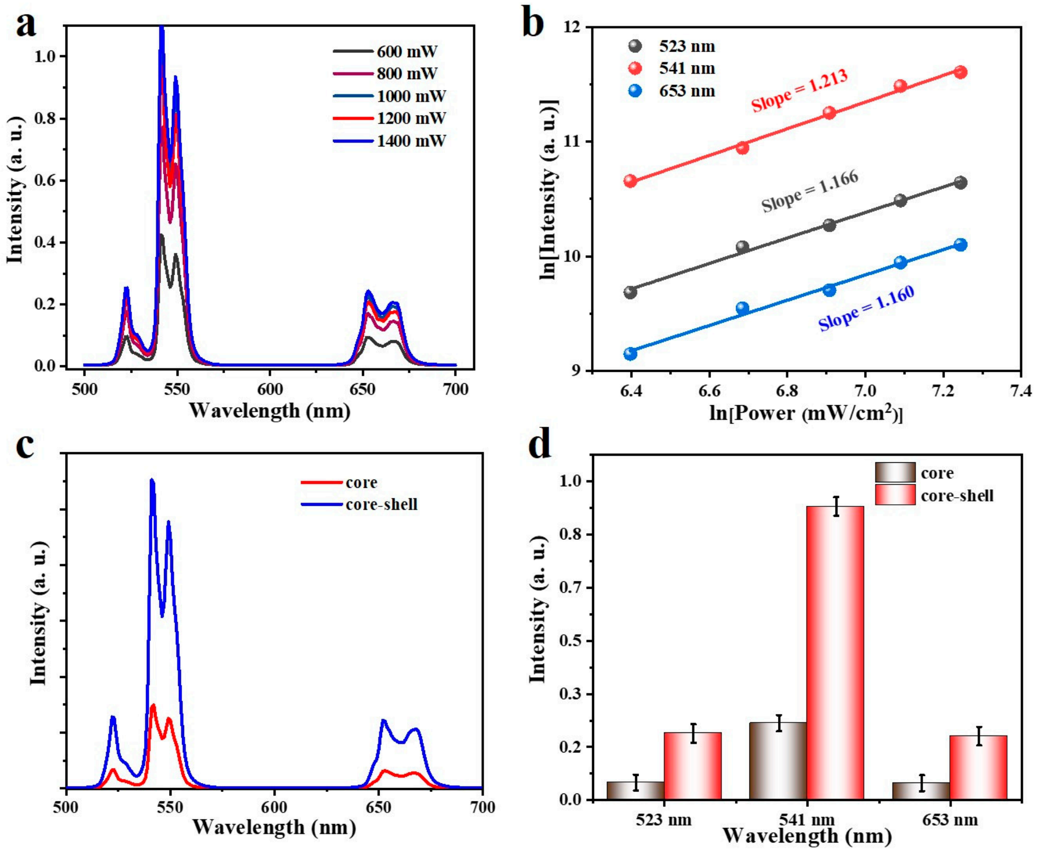

Figure S3. (a) Upconversion luminescence spectra in visible regions of nanocrystals excited at different power densities of 980 nm. (b) The relationship between emission intensity and excitation power. (c,d) Comparison of upconversion luminescence intensity between core and core-shell nanoparticles.

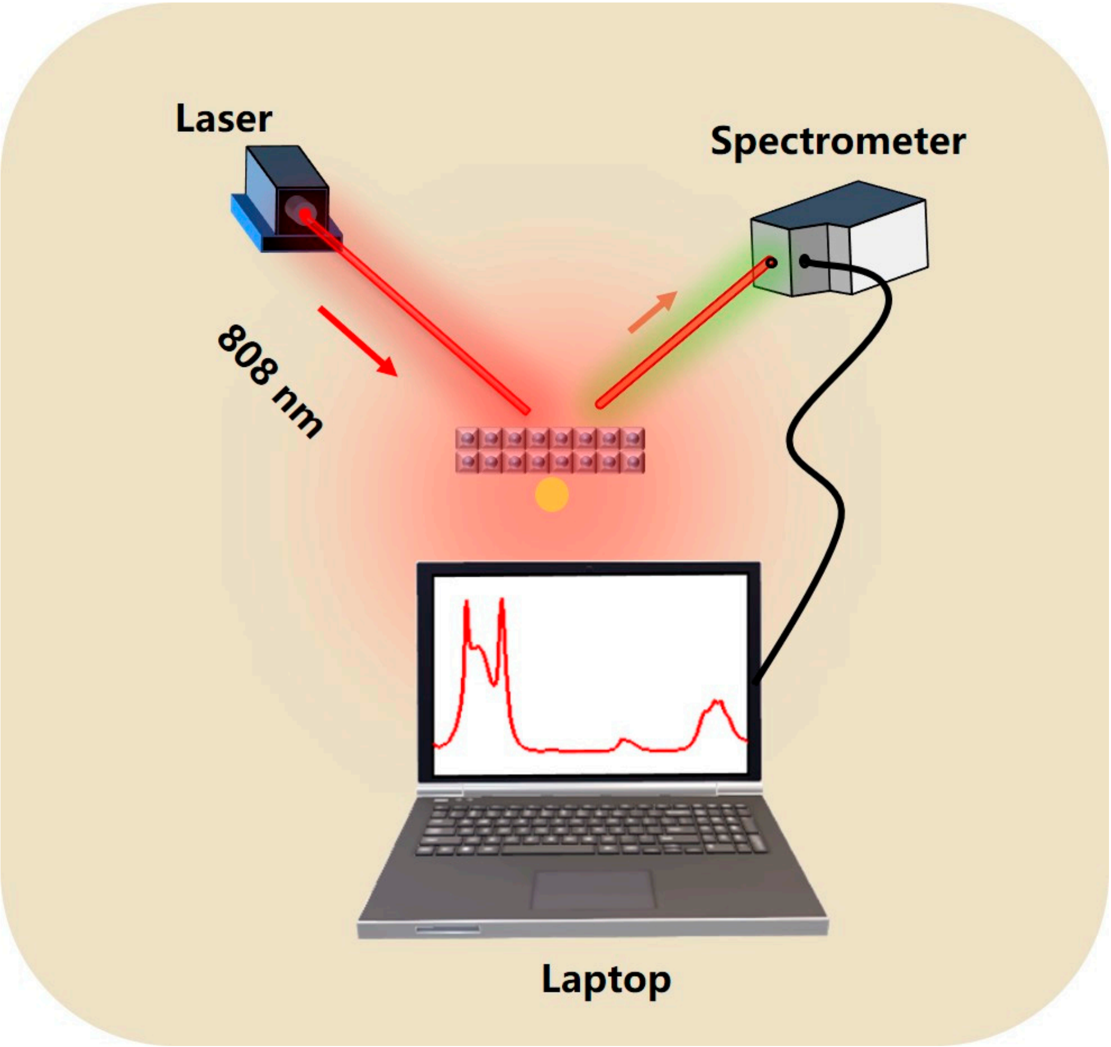

Figure S4. Diagram of the non-contact temperature measuring system.

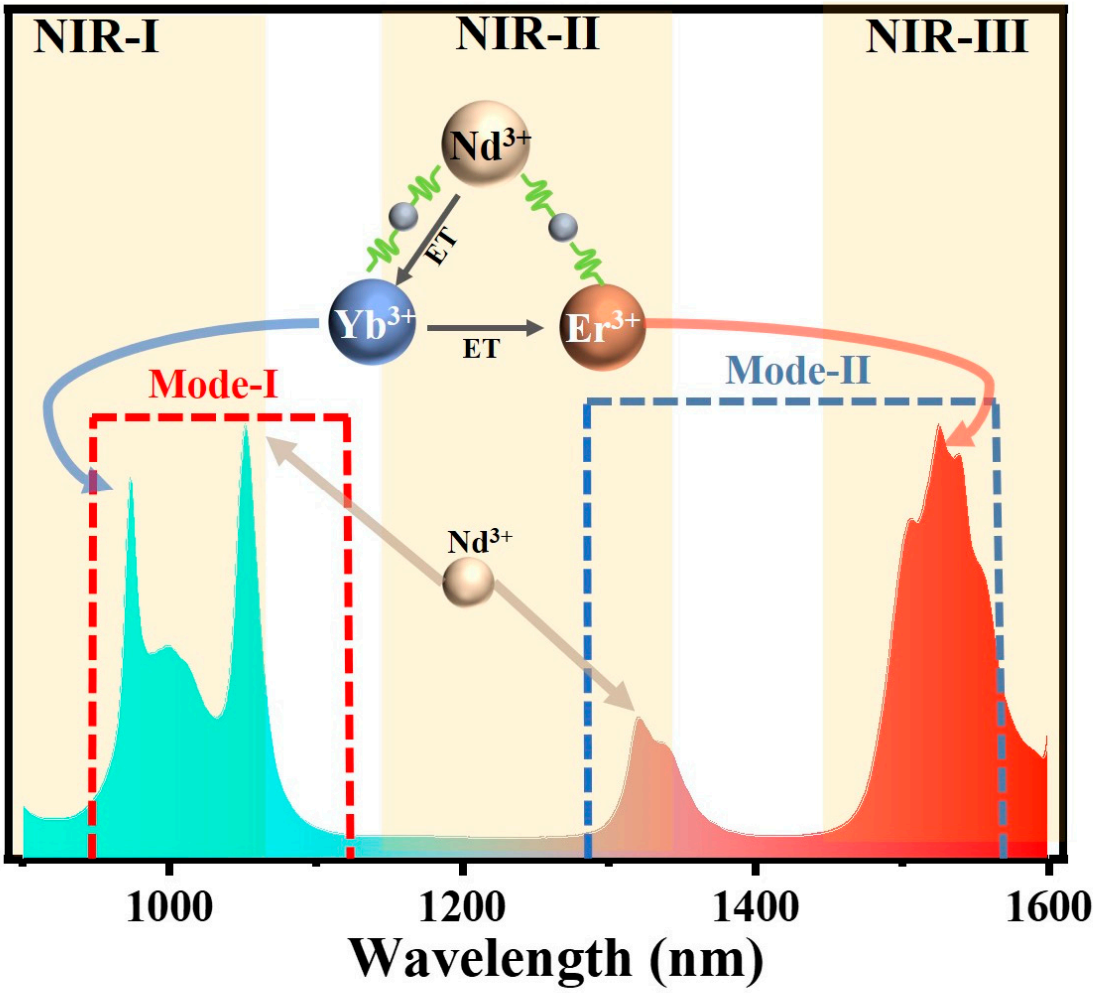

Figure S5. Schematic diagram of multi-mode temperature measurement principle.
